# Effect of tobacco on periodontal disease and oral cancer

**DOI:** 10.18332/tid/106187

**Published:** 2019-05-09

**Authors:** Yixin Zhang, Jinxiu He, Bing He, Ruijie Huang, Mingyun Li

**Affiliations:** 1 State Key Laboratory of Oral Diseases, West China School of Stomatology, Sichuan University, Chengdu, China; 2 Protein Section, Laboratory of Metabolism, Center for Cancer Research, National Cancer Institute, National Institutes of Health, Bethesda, United States

**Keywords:** tobacco, periodontal disease, oral cancer, inflammation, alveolar bone

## Abstract

**INTRODUCTION:**

Periodontal disease and oral cancer are common health hazards. Epidemiological investigations show that smoking, periodontal disease and oral cancer are closely related. Tobacco is one of the major risk factors for periodontitis and oral cancer.

**METHODS:**

A systematic literature review was performed. To identify relevant studies, the following online databases were searched using specific keywords: PubMed, Web of Science and CNKI.

**RESULTS:**

Tobacco not only possesses an addictive effect, but it aggravates periodontal disease by promoting the invasion of pathogenic bacteria, inhibiting autoimmune defense, aggravating the inflammatory reaction, and aggravating the loss of alveolar bone. According to current evidence, tobacco significantly aggravates the development and progression of periodontal disease and oral cancer, and periodontal disease may be related to the prevalence of oral cancer.

**CONCLUSIONS:**

Clinicians should strongly recommend that smokers undertake a strategy to stop smoking to avoid the exacerbation of nicotine-related periodontal disease and to reduce the incidence of oral cancer.

## INTRODUCTION

Cigarette smoking is a well-established risk factor for periodontal disease and it is the strongest factor among the modifiable ones^1^. Research evidence suggests that smokers have a higher tendency to problems such as teeth and bone loss and gingival recession compared to non-smokers, and to the formation of periodontal pockets, which increase the probability to suffer from more severe periodontal disease^2-5^. Tobacco can affect the function and proliferation of periodontal cells such as gingival fibroblasts, periodontal membrane cells, periodontal ligament cells and other cells, thus inducing cell apoptosis. It can also affect the invasion of periodontal disease, inhibit the autoimmune defense, and aggravate the inflammation reaction to damage and destroy the alveolar bone. Oral cancer is a common health hazard, which is also closely related to tobacco. This article reviews the relationship among tobacco, periodontal disease and oral cancer.

## METHODS

PubMed, Web of Science databases and CNKI were selected as the primary databases. Searches were conducted by crossing keywords ‘tobacco’ and ‘nicotine’ separately with ‘periodontal disease’ and ‘oral cancer’. Searches were limited to studies in English and published from 1947 to 2019. Inclusion criteria were: articles focused on tobacco and nicotine and focused on any of the six key themes (periodontal disease, oral cancer, inflammation, alveolar bone, periodontal cells, and oral bacterial species) according to the objective of this review. Firstly, titles and abstracts were assessed, and then articles were included or excluded based on their relevance. In this review, clinical, microbiological and immunological data regarding tobacco, periodontal disease and oral cancer were collected, compared, analyzed and studied, and the relationship among them was summarized.

## RESULTS and DISCUSSION

### Clinical findings

Periodontal disease is a common chronic inflammatory disease that causes tooth loss in adults, and it is characterized by the destruction of the supporting structures of teeth including the gingiva, cementum, periodontal ligament, and alveolar bone^6,7^. It is now recognized that periodontal disease is a multifactorial disease in which plaque is the triggering factor of periodontal disease. Common risk factors for periodontal disease include gender, poor lifestyle such as smoking habit and alcohol consumption, certain systemic diseases such as diabetes, prediabetes, obesity and metabolic syndrome, and genetic factors^8^. The first observation on the relationship between smoking and periodontal tissues occurred in the1940s, when Pindborg^9,10^ demonstrated that necrotizing ulcerative gingivitis was associated with tobacco consumption. Several epidemiological studies clearly demonstrated a strong association between tobacco use/smoking habit and periodontal diseases in diverse populations^11-13^. In general, evidence indicates that smokers have more severe periodontal diseases, with increased bone attachment and tooth loss, gingival recession, and pocket formation compared to non-smokers and there is a dose-response relationship between the number of cigarettes smoked per day and odds of periodontal disease^14,15^. The dangers of passive smoking have also been emphasized as some evidence linked passive smoking to diseases and death in non-smokers^16^.

Oral cancer is a common health hazard, with approximately 300378 new patients and 2.7 per 100000 mortality worldwide in 2012; the incidence increased in young and middle-age population groups in recent years^1,17,18^. According to previous reports, oral cancer is the world’s sixth most common cancer and squamous cell carcinomais the most prevalent among oral malignancies^19^. Oral cancer is considered as a multi-factor disease, in which tobacco, alcohol and betel quid are the major risk factors^17^. Smoking has a significant epidemiological correlation with oral cancer and plays an important role in its occurrence and development. Smokers are 7 to 10 times more likely to develop oral cancer and 3 times more likely to develop a second primary cancer than non-smokers^20,21^. In addition, some studies reported a positive correlation between periodontal disease and oral cancer, suggesting that periodontal disease is an independent risk factor for oral cancer, and smoking promotes this correlation^22-26^.

### Effect of tobacco on periodontal cells

Periodontal ligament cells (hPDLCs) are the main cell components in the periodontal membrane, and they have chemotactic adhesion, proliferation, biosynthesis and differentiation into cementite and osteoblasts. Various tissues, such as cementum and alveolar bone, are formed, playing an important role in the maintenance, regeneration and repair of tooth support tissues. Table 1 summarizes the effect of tobacco on periodontal cells.

**Table 1:** The effect of tobacco on periodontal cells

*Reference*	*Type of study*	*Microbiological technique*	*Principal findings*
**Lallier et al.** **^29^**	*In vitro*	Fluorescent microscopy	With increasing concentrations of nicotine HGFs and PDLFs reduce cell viability
**Yang et al.** **^30^**	*In vitro*	Transmission electron microscope and immunofluorescence observations	Nicotine increases autophagy level of hPDLCs
**Zhang et al.** **^31^**	*In vitro*	RT-PCR	CSE increases the collagen-degrading ability of hGFs
**Zhou et al.** **^32^**	*In vitro*	RT-PCR	Nicotine and P. gingivalis had an additive effect on human gingival fibroblast-mediated collagen degradation
**Deveci et al.** **^33^**	*In vitro*	Light microscope	Nicotine disrupts periodontal membrane and prevents tooth to anchor in dental alveoli by disrupting epithelial structure
**Park et al.** **^34^**	*In vitro*	RT-PCR	The effect of nicotine and lipopolysaccharide on hGFs up-regulation of SIRT1 mRNA plays an anti-inflammatory and pro-inflammatory effect
**Brejc et al.** **^36^**	*In vitro*	RT-PCR	Nicotine alters periodontal cells directly via nAChRs
**Moga et al.** **^38^**	*In vitro*	Histological examinations	Nicotine exerts dose- and time-dependent cytotoxic effects
**Ng et al.** **^39^**	*Clinical*	Cell migration analysis and gene expression analysis	Cigarette smoking reduces proliferation rate and retarded migration capabilities of PDLSC

Many of the underlying effects of tobacco products on periodontal tissues may be due to a direct inhibition of normal fibroblast function. Both gingival fibroblasts (hGFs) and periodontal ligament fibroblasts (PDLFs) display reduced cell viability with increasing concentrations of cigarette smoke extract (CSE) and nicotine^27,28^. PDLFs are also more sensitive to nicotine compared with hGFs^28^. Du et al.^29^ indicated that nicotine activates the autophagy of hPDLCs by increasing the number of autophagosomes and by the up-regulation of the autophagy related protein LC3 expression, suggesting that nicotine can increase autophagy of hPDLCs, thus affecting the occurrence and development of smoking related periodontal disease.

In addition to the direct damage to cells, a study shows that the combination of CSE increases the collagen-degrading ability of hGFs^30^. Nicotine, as the main active ingredient in tobacco, increases human gingival fibroblast-mediated collagen degradation, in part through the activation of membrane-associated matrix metalloproteinases (MMPs). Indeed, MMP-14 and MMP-2 produced by the nicotine-treated human gingival fibroblasts undergo more readily zymogen activation. Nicotine has an additive effect on human gingival fibroblast-mediated collagen degradation when combined with the presence of *P. gingivalis* ^31^. Deveci et al.^32^ found that nicotine reduces the production of MMPs, disrupts collagen synthesis and causes periodontitis in rats. They observed that nicotine increases periodontitis by disrupting the periodontal membrane and prevents the anchorage of teeth in the dental alveoli by disrupting the epithelial structure. In addition, the action of nicotine and lipopolysaccharide on SIRT1 mRNA up-regulation in hGFs has an anti-inflammatory and pro-inflammatory effect^33^. Studies show that nicotine may alter periodontal cells directly via nAChRs, leading to pathophysiological effects and the development of tobacco-related diseases in these cells^34-36^.

Cigarette smoking contributes to the development of destructive periodontal diseases and delays the healing process. Moga et al.^37^ isolated four types of cells from human periodontium: gingival ligament stem cells (GLSCs), periodontal ligament stem cells (PDLSCs), gingival tissue stem cells (GTSCs), and alveolar bone stem cells (ABSCs), aiming at assessing the cytotoxic effect of nicotine on periodontal mesenchymal stem cells. They found that nicotine exerts dose- and time-dependent cytotoxic effects. Ng et al.^38^ hypothesized that the delayed healing process in cigarette smokers is caused by the effected regenerative potential of smoker PDLSC. They cultured PDLSC from teeth extracted from smokers and non-smokers, and they found significantly reduced proliferation rate and retarded migration ability in smoker PDLSC.

### Effect of tobacco on bacteria

Generally, smokers have more calculus deposit than non-smokers, and the calculus from smokers is stiffer and more tightly attached to teeth than that from non-smokers. Acquired pellicle attaching to a tooth surface is the initial step for dental biofilm formation, followed by bacterial cell attachment to the acquired pellicle^39^. *In vitro* experiments showed that lower concentrations of nicotine can stimulate oral biofilm formation and influence cell metabolism of biofilm microorganisms^40^. Unfortunately, there are still questions regarding the ability of nicotine in increasing the metabolic activity of microorganisms in the oral cavity.

Some studies reported no differences in the prevalence of subgingival species between smokers and non-smokers with periodontitis^41,42^. Cogo et al.^43^ performed a study to evaluate the effects of nicotine, cotinine, and caffeine on the viability of some oral bacterial species, and their findings indicate all these compounds in the concentrations used, cannot significantly affect the growth of the oral bacterial strains considered. Moreover, the species considered in their study appear not to metabolize these compounds.

However, some authors showed that smoking increases the prevalence and amount of some oral bacterial strains^44^. Table 2 is a summary of some of the results attesting the factors that increase the bacterial invasiveness due to the presence of tobacco. *Streptococcus gordonii (S. gordonii)* plays a central role in initiating dental biofilm formation, and in providing binding sites for later colonizers such as *P. gingivalis*, allowing its attachment and the generation of mature biofilm. Huang et al.^45^ found that nicotine stimulates *S. gordonii* planktonic cell growth, biofilm formation, aggregation, and gene expression translated into binding proteins. Furthermore, Shan et al.^46^ suggested that nicotine can enhance the growth of *Candida albicans (C. albicans)* and *C. parapsilosisin vitro* and influence their adherence to the surface of microplate wells that mimic the tooth surface adherence. These effects may promote later pathogen attachment to tooth surfaces, the accumulation of tooth calculus, and the development of periodontal disease in cigarette smokers.

**Table 2:** Tobacco increases bacterial invasiveness

*Reference*	*Type of study*	*Microbiological technique*	*Principal findings*
**Haffajee et al.** **^45^**	Clinical	PCR	Smoking increases the likelihood and proportion of certain microbial epidemics
**Huang et al.** **^46^**	*In vitro*	RT-PCR	Nicotine stimulates *S. gordonii* planktonic cell growth, biofilm formation, aggregation, and gene expression of binding proteins
**Shan et al.** **^47^**	*In vitro*	RT-PCR	Nicotine enhances the growth of *C. albicans* and *C. parapsilosis* and influences their adherence
**Cogo et al.** **^49^**	*In vitro*	Colony-forming unities	Nicotine and cotinine interfere with *P. gingivalis* ability to associate and invade the epithelial cells
**Liu et al.** **^50^**	*In vitro*	Absorption photometry	Small doses of nicotine and mecamylamine can increase the biofilm formation of the gingival porphyrins and associated actinobacteria
**Cogo et al.** **^51^**	*In vitro*	Liquid chromatography-mass spectrometry and primary sequence	Exposure to nicotine and cotinine, the proteome of *P. gingivalis* changes

*P. gingivalis* is an important colonizer of the subgingival crevice and is a major pathogenic agent in the initiation and progression of severe forms of periodontal disease. Moreover, it is found in significantly higher numbers in smokers than in non-smokers, and infection is more persistent in smokers compared to non-smokers^47^. With this in mind, the consequences of the presence of *P. gingivalis* are discussed separately. Cogo et al.^48^ performed a study indicating that cotinine and nicotine interfere with the ability of *P. gingivalis* to invade the epithelial cells. High doses of CSE and nicotine inhibit bacterial growth, while low doses can increase the biofilm formation of the gingival porphyrins and associated actinobacteria, as confirmed by previous reports^40,49^. Cogo et al.^50^ successfully identified the expression of different proteins by liquid chromatography mass spectrometry and main sequence database of the mascot search engine, and used the DAVID tool to carry out the genetic ontology. Their results revealed the changes in the proteome of *P. gingivalis* following exposure to nicotine and cotinine, suggesting that these substances may modulate, with minor changes, protein expression. However, Baek et al.^51^ suggested that nicotine did not significantly affect total protein expression. It cannot be excluded that a significant change in the protein profile actually occurred, but it was not detectable by visual inspection of gel electrophoresis.

Considering the limitations of the current study, it can only be concluded that nicotine has the potential to elicit a stress reaction, and therefore may serve as an environmental modulating factor for bacterial metabolism and survival.

### Tobacco reduces periodontal tissue immune defense

The multiple defence barriers of the periodontal tissue are represented by the epithelial barrier, immune cells, saliva and gingival fluid. This mechanism plays an important role in the persistence of dental plaque in the gingival furrow and the protection of periodontal tissue from bacterial invasion and destruction. Table 3 summarizes the mechanisms by which tobacco can reduce the immune defense of periodontal tissues.

**Table 3:** Tobacco reduces periodontal tissue immunity

*Defense barriers of periodontal tissue*	*Reference*	*Microbiological technique*	*Principal findings*
**Epithelial barrier**	Bondy-Carey et al.^53^	Bacterial culture and cytokine profiling	CSE-exposure promotes *P. gingivalis* survival and invasion by reducing the pro-inflammatory cytokine burden
	Imamura et al.^55,56^	MAPK and PCR	Nicotine exerts effects on the migration of human gingival epithelial cells through the activation of the MAPK ERK1/2 and p38 signaling pathways
**Immune cells**	White et al.^57^	Fluorescence-based assays and PCR	CSE reduces speed, velocity and directionality relative to untreated neutrophils
	Erdemir et al.^58^	Cell culture	Tobacco effects the secretion of cytokines and inflammatory mediators from immune cells, such as neutrophils and mononuclear cells
	Ge et al.^60^	Cell culture and cytokine and chemokine detection	Nicotine promotes PDL cell–CD4+T cell-mediated inflammatory response and matrix degradation
	Yanagita et al.^61^	Cell culture and cytokine and chemokine detection	DCs in nicotine culture impair T-cell proliferation and reduced host immunity
**Gingival crevicular fluid**	An et al.^62^	Enzyme-linked immunosorbent assay	Nicotine causes vascular endothelial cells to contract, reducing blood flow, and reducing the immune cells and GCF
	Bozkurt et al.^63^	Enzyme-linked immunosorbent assay	Tobacco reduces leptin levels in GCF in periodontitis
	Bunaes et al.^65^	Checkerboard DNA-DNA hybridization	Smoking increases the cytokine and inflammatory in GCF and aggravates periodontitis

Epithelial cells are recognized as the first line of defence against bacterial infection and environmental harmful stimuli such as cigarette smoke (CS). Although previous studies explored the effects of nicotine on host cells, mechanisms used by CS to affect cellular functions remain uncertain. *P. gingivali*s alone induces low levels of IL-1β and IL-8 on epithelial cells, but high levels of both cytokines are produced with the addition of neutrophils. CSE exposure reduces the pro-inflammatory cytokine burden, which may promote *P. gingivalis* survival and invasion^52^. Moreover, cell migration and proliferation are key aspects of many biological processes, including wound healing and tissue regeneration^53^. During the wound healing process, epithelial cells at the wound edges start to migrate and proliferate to cover the denuded area. This cell migration is necessary for re-epithelialization. The Imamura et al.^54^ study demonstrated that low concentrations of CSE increased the invasion of human gingival epithelial cells infected with *P. gingivalis* and induced changes in cytoskeleton and integrin expression, thereby modulating cell migration. Furthermore, they found that nicotine in CSE exerts effects on the migration of human gingival epithelial cells through the activation of the MAPK ERK1/2 and p38 signaling pathways^55^.

It has been reported that neutrophils pretreated with CSE exhibited reduced speed, velocity and directionality compared to untreated neutrophils^56^. In addition, tobacco affects the secretion of cytokines and inflammatory mediators from immune cells such as neutrophils and mononuclear cells. Tobacco may impair the chemotaxis and phagocytosis of neutrophils, modifying the production of cytokines or inflammatory mediators^57^. The functional roles of T cells in periodontal disease lesions remain to be elucidated. In human studies, T cells infiltrating gingival lesions expressed mRNA for T-helper (Th)1/Th2 and for regulatory cytokines^58^. Nicotine deteriorates periodontal disease partially by promoting PDL cell–CD4+T cell-mediated inflammatory response and matrix degradation^59^. On the other hand, *P. gingivalis* LPS-stimulated monocyte-derived dendritic cells induced increased proliferation with increasing dendritic cells (DC) number. However, DCs cultured in the presence of nicotine significantly impair T-cell proliferation and reduce host immunity^60^. DCs are key mediators between innate and adaptive immunity, and they stimulate naive T cells to differentiate to effector T-cell subsets that may be actively involved in the immunopathogenesis of periodontal diseases.

Other effects of smoking are the decrease in blood flow and impairment of revascularization in the periodontal tissues, thereby causing delayed wound healing. Nicotine causes the contraction of vascular endothelial cells^61^, reducing blood flow, making gingivitis less clinically evident and easy to be missed, reduces the immune cells and gingival crevicular fluid (GCF), and may reduce the host immunity during early periodontitis. Leptin emerges as a pleiotropic molecule involved in several physiological and pathological conditions, and higher leptin levels in healthy sites in periodontitis patients may play a protective role against periodontal disease. However, Bozkurt et al.^62^ results suggest that tobacco reduces leptin levels in GCF in periodontitis. Monocyte chemoattractant protein-1 (MCP-1) is involved in the activation and recruitment of inflammatory and immune cells to infected sites, thereby mediating a variety of pathophysiological conditions. Estimation of serum and GCF MCP levels can be a reliable indicator of periodontal disease activity. The Sukumaran et al.^63-65^ study concluded that smoking increases GCF MCP levels, cytokine and inflammatory factors, thus aggravating periodontitis. This last aspect is described in detail in the next section.

### Tobacco exacerbates periodontal tissue inflammatory response

Smoking not only alters the host response, including vascular function, and neutrophil/monocyte activity, but also increases adhesion molecule expression, antibody production, and cytokine and inflammatory mediator release^66^. Table 4 shows a summary of the mechanisms by which tobacco aggravates periodontal inflammation. The mechanism of immune inflammation that can accurately explain the aggravation and progression of periodontal disease with nicotine has not been fully elucidated. Nevertheless, oxidative stress and changes in the immune inflammatory system seem to play an important role.

**Table 4:** Tobacco exacerbates periodontal tissue inflammatory response

*Reference*	*Microbiological technique*	*Principal findings*	*Effect on periodontal tissue*
**Deveci et al.** **^33^**	Immunohistochemical staining	Up-regulate MMP-1, MMP-2 and MMP-3 gene expression in arterial smooth muscle cells, vascular endothelial cells, periodontal ligament fibroblasts and osteoclasts	MMPs are associated with periodontal tissue destruction, which mainly degrade extracellular matrix molecules such as collagen, gel, and elastin
**Meenawat et al.** **^68^**	PCR	Association of smoking status with periodontal destruction is correlated with the increased mRNA expression of IL-1β in chronic periodontitis patients	Cytokines cause inflammatory responses that exacerbate periodontal tissue destruction
**Johnson et al.** **^69^**	ELISA	Nicotine promotes the secretion of IL-1, IL-6, IL-8, TNF and McP-1 by periodontal cells, such as gingival keratinocytes and hGFs	
**Xanthoulea et al.** **^71^**	ELISA	Nicotine injection affected inflammatory mediators like TNF, IL-6 and IL-12 while it induced a down-regulation in the expression of VEGF, PDGF, TGF-β1 and TGF-β2, IL-10	Reduced growth factor expression by nicotine might contribute to the overall detrimental effects of tobacco use in wound healing
**Cho et al.** **^73^**	RT-PCR	Nicotine and LPS synergistically induced the production of COX-2 and PGE2 and increased the protein expression of JAK/STAT	PGF2 and COX-2 are associated with bone loss in periodontitis, inducing MMPs and osteoclast absorption

Proteases from host and microorganism play an important role in periodontitis tissue destruction. Proteases break down proteins by hydrolyzing peptides. MMPs are a family of proteolytic enzymes associated with periodontal tissue destruction, which mainly degrade extracellular matrix molecules such as collagen, gel, and elastin^31^. Nicotine, which is the major ingredient of cigarette smoke, up-regulates MMP-1, MMP-2 and MMP-3 gene expression in arterial smooth muscle cells, vascular endothelial cells, periodontal ligament fibroblasts and osteoclasts. Nicotine administration also causes a change in fibroblast activity due to the change of collagen fiber synthesis and decreased MMP2 expression^32^.

Cytokines are proteins secreted by cells that act as information molecules to transmit signals to other cells. Cytokines have many roles, including initiation and maintenance of immune and inflammatory responses, regulating cell growth and differentiation. The two cytokines, interleukin (IL) and tumor necrosis factor (TNF), play an important role in periodontal tissue destruction. Association of smoking status with periodontal destruction can thus be correlated with the increased mRNA expression of IL-1β in chronic periodontitis patients^67^. A number of studies demonstrated that nicotine differentially regulate IL-1, IL-6, IL-8, TNF and MCP-1 production by periodontal cells, such as gingival keratinocytes and hGFs^68,69^. Nicotine injection in excisional full-thickness skin wounds minimally affects inflammatory mediators such as TNF, IL-6 and IL-12, while it induces a down-regulation in the expression of growth factors such as VEGF, PDGF, TGF-β1 and TGF-β2, and the anti-inflammatory cytokine IL-10^70^. Reduced growth factor expression by nicotine might contribute, at least in part, to the overall detrimental effects of tobacco use in wound healing and skin diseases.

Studies suggest that LPS and nicotine synergistically induce the production of cyclooxgenase-2 (COX-2) and prostaglandin E2 (PGE2) and increase JAK/STAT protein expression. Treatment with a JAK inhibitor blocks the production of COX-2 and PGE2 and the expression of pro-inflammatory cytokines, such as TNF-α, IL-1β, and IL-6 in LPS- and nicotine-stimulated osteoblasts^71,72^. PGE2 is associated with bone loss in periodontal disease, inducing MMPs and osteoclast absorption.

### Effect of tobacco on the alveolar bone

Smoking produces an adverse effect on clinical periodontal variables and alveolar bone height and density, acting as a potential risk factor for alveolar bone loss^73,74^. These observations highlight the destruction of periodontal tissue by smoking and the unfavorable clinical course of periodontal disease in patients with a cigarette smoking habit. Table 5 summarizes the effect of tobacco on the alveolar bone.

**Table 5:** The effect of tobacco on the alveolar bone

*Reference*	*Microbiological technique*	*Principal findings*
**Kubota et al.** **^84^**	RT-PCR	CSE and Nicotine increased the number of osteoclasts and upregulated the expression of receptor activator of nuclear factor κB ligand
**Wu et al.** **^85^**	ELISA	Nicotine has the ability to act on osteoclast precursors, inducing its osteoclastogenic differentiation
**Costa-Rodrigues et al.** **^86^**	RT-PCR	In the presence of the growth factors, the osteoclastogenesis enhancers M-CSF and RANKL a significant increase in their resorbing ability is also achieved
**Saito et al.** **^88^**	Micro-CT	Nicotine can inhibit osteoblast differentiation
**Katono et al.** **^89^**	Immunohistochemical Staining	Culture of nicotine and osteoblasts Saos-2 Cells significantly increase in the expression of MMP
**Tura-Ceide et al.** **^92^**	RT-PCR	Tobacco is associated with lower numbers of circulating stem cells, which affects the homing and functional capabilities of bone marrow-derived mesenchymal stem cells

Smoking alters alveolar bone metabolism and therefore can synergistically act on alveolar bone loss^75^. Hapidin et al.^76^ showed that nicotine significantly decreased the trabecular bone volume, trabecular thickness, double-labeled surface, mineralizing surface, mineral appositional rate, and bone formation rate, while causing an increase in the single-labeled surface, osteoclast surface, and eroded surface. Eratilla et al.^77^ found that nicotine can cause alveolar bone absorption and vascular dilatation, hemorrhage, periodontal degeneration, hyaluronosis, and necrosis. Nicotine also affects bone remodeling during orthodontic movement, reducing angiogenesis, osteoclast-like cells and Howship’s lacunae, thereby delaying the collagen maturation process in developed bone matrix^78,79^. Furthermore, cigarette smoking has a detrimental effect on early bone tissue response around sandblasted acid-etched implant surface topographies and narghile smoking increases peri-implant soft-tissue inflammation and crestal bone loss^80-82^. Overall, tobacco accelerates the loss of alveolar bone and has a negative effect on the remodeling of alveolar bone during implantation and orthodontic treatment proportional to the dose of CSE and nicotine.

Osteoclast and osteoblast play a key role in bone resorption caused by periodontal disease. Tooth loss is mainly a result of alveolar bone resorption, which reflects an increased osteoclast formation and activation. Kubota et al.^83^ reported that the systemic administration of cigarette smoke condensate or nicotine increases alveolar bone loss. Concomitantly, the number of osteoclasts in periodontal tissues increases and the expression of receptor activator of nuclear factor κB ligand is up-regulated. Osteoclast formation in periodontal tissue is a multi-step process driven by osteoclastogenesis, supporting cells such as human periodontal ligament cells and CD4+ T cells, and inflammatory cytokines that induce osteoclastogenesis^84^. Thus, nicotine at levels found in the plasma of the smokers, has the ability to act directly on osteoclast precursors, inducing its osteoclastogenic differentiation, while in the presence of growth factors such as inflammatory cytokines, the osteoclastogenesis enhancers M-CSF and RANKL induce a significant resorbing increase^85^. Nicotine, which is the major ingredient of cigarette smoke, can also inhibit osteoblast differentiation^86,87^. Katono et al.^88^ found a significant increase in the expression of MMP after culture of nicotine and osteosarcoma Saos-2 possessing several osteoblastic features. MMP-2 levels were higher in the exposed rats compared with the non-exposed rats, suggesting that MMP may be one of the molecules responsible for the increased tissue degradation observed in the periodontal tissues of smokers. MMP is considered as an important factor in periodontal tissue damage because of strong matrix degradation ability. Nicotine may accelerate the metabolic rate of the bone matrix by increasing the formation of MMP, which can affect osteoclast migration and adhesion and cause osteoblastic apoptosis, there by destroying the dynamic balance of bone formation and absorption.

A number of experimental animal studies and *in vitro* studies confirmed that nicotine impairs bone healing, diminishes osteoblast function, causes autogenous bone graft morbidity, and decreases graft biomechanical properties^87,89^. Some studies show that CSE and nicotine are also associated with a lower number of circulating stem cells and may severely affect their mobilization, trafficking and homing, consequently affecting the homing and functional capabilities of bone marrow-derived mesenchymal stem cells that are closely related to alveolar bone healing^90,91^. De Campos et al.^92^ aimed at evaluating the influence of smoking on gene expression of molecules related to bone metabolism in alveolar bone tissue from sites designed to receive dental implants. Multiple regression analysis indicated that smoking negatively affects mRNA expression of bone sialoprotein and osteocalcin and positively alters the expression of type I collagen despite age, gender, and dental arch. These results support the hypothesis that some bone markers in alveolar tissue are modulated by smoking, thus explaining the negative impact of smoking on bone healing.

### Effect of tobacco on tooth germ

The negative effects of tobacco on periodontal disease may begin earlier than we thought, during tooth germ development. Saad et al.^93^ proposed that nicotine, or its metabolic byproducts, lead to retarded, less differentiated, and breadth reduced developing molars of the experimental mice fetuses in comparison with controls with no nicotine treatment. Wang et al.^94,95^ studied the effect of nicotine on dental germ development of mouse molar *in vitro*, and they found that nicotine inhibits the secretion of bone morphogenetic protein (BMP) in dental germ of mouse and dental papilla mesenchymal cells. They found also a reduced volume of developing tooth germ, a lower number and rapidity of odontoblast proliferation compared to the group with no nicotine treatment, with a decreased or inhibited formation of predentin.

Tobacco and its metabolic byproducts interfere with the normal interaction between epithelial and mesenchymal components of the developing tooth, having teratogenic effects on the tooth, becoming a local factor promoting the occurrence and development of periodontal disease. On the other hand, smoking causes a reduction in tooth germ number, cell proliferation inhibition and differentiation, and reduced tooth number resulting in a potential negative effect on the prognosis of periodontal disease. An epidemiological study in Japan suggested that, maternal smoking in the first trimester of pregnancy is significantly associated with an increased prevalence of dental caries in children compared with children from non-smoking mothers during pregnancy^96^. However, epidemiological studies of the association between smoking exposure and periodontal disease during pregnancy are limited.

### Relationship between tobacco, periodontal disease and oral cancer

Oral cavity cancer is a debilitating and often fatal cancer closely linked to tobacco product use. Smoking has a significant epidemiological correlation with oral cancer and plays an important role in its occurrence and development. Smokers are 7 to 10 times more likely to develop oral cancer and 3 times more likely to develop a second primary cancer than nonsmokers^20,21^. The devastating link between tobacco products and human cancers results from a powerful alliance of two factors — nicotine and carcinogens. Nicotine is addictive and toxic, but there is no scientific evidence that nicotine is a carcinogen, and nicotine is not classified as a carcinogen by the IARC. However, this addiction causes people to use tobacco products continually, and these products contain many carcinogens. There are more than 60 carcinogens in cigarette smoke and at least 16 in unburned tobacco. Among these, tobacco-specific nitrosamines such as 4-(methylnitrosamino)-1-(3-pyridyl)-1-butanone (NNK) and N’-nitrosonornicotine (NNN), polycyclic aromatic hydrocarbons (such as benzoapyrene) and aromatic amines (such as 4-aminobiphenyl) seem to play an important role as causes of oral cancer^97^.

Recently, several studies reported the relationship between periodontal disease and oral cancer. Some studies reported a positive association between periodontal disease and oral cancer after adjustment for potential confounders for several factors including age, gender, smoking status, and alcohol consumption, and suggested that periodontal disease is an independent risk factor for oral cancer, while smoking modifies this association^22-26^. However, other studies reported no association between periodontal disease and oral cancer^98^. The conclusion regarding the relationship between periodontal disease and oral cancer is still controversial. A systematic review by Javed^99^ suggested a correlation between periodontal disease and oral tumors, and this association changes after adjusting for confounding factors such as tobacco and alcohol. Prior to this work, some other reports also agree with this view^100,101^. In addition, many published meta-analyses are available that illustrate this problem. Some of the published meta-analyses in recent years are listed in Table 6 ^102-104^. It is noteworthy that some of the periodontal disease risk factors, particularly tobacco and betel quid use, have also been linked with the etiology of oral cancer. Although in some small studies, tobacco or alcohol use and socioeconomic confounders attenuate the association between periodontitis and oral cancer, overall findings of a recent systematic review concluded that periodontitis is likely to be associated with increased head and neck cancer risk^105^.

**Table 6:** Oral cancer is associated with periodontal disease

*Reference*	*Sample*	*OR*	*95% CI*	*Conclusion*
**Ye et al.** **^103^**	11 case-control studies	3.21	2.25–4.16 p<0.05	Periodontal disease can increase the oral cancer risk by nearly 2-fold
**Yao et al.** **^104^**	5 case–control studies	3.53	1.52–8.23 p=0.003	Patients with periodontal disease have increased susceptibility to oral cancer
**Zeng et al.** **^105^**	2 cohort and 6 case-control studies	2.63	1.1.68–4.14 p<0.001	Based on currently evidence, PD is probably a significant and independent risk factor of HNC

In summary, there is only scarce evidence that periodontal disease may be related to the prevalence of oral cancer. Nevertheless, the association of periodontal disease to oral neoplasms may be explained indirectly by the possibility that a broken mucosal barrier in the presence of periodontal disease could lead to a subsequent penetration of carcinogens such as tobacco and alcohol. The association between periodontitis and oral cancer does not rule out a link between tobacco and oral cancer, and further investigation is warranted.

A smoker absorbs half a milligram of nicotine in each cigarette, which is degraded to cotinine (the major metabolite close to nicotine), nicotine glucuronide, nircotrine and nornicotine primarily^106^. Nicotine possesses a very short half-life in the blood, approximately 2 h, while cotinine, a major metabolite of nicotine with a longer half-life (17 h vs 30 min), may be a more useful biochemical marker of smoking status^107^. Cotinine has been widely used as a stable biomarker of tobacco exposure and has been used to correlate its levels with periodontal disease severity^108,109^.

It should be made clear that most of the findings described are from experimental studies in cell cultures and not from human studies. Some experimental studies used exposure to CSE, but others have used nicotine instead of tobacco. Although nicotine is the major ingredient of cigarette smoke, what tobacco does to human health cannot be attributed to nicotine alone as there are so many molecules contained in tobacco smoke. Tobacco metabolism in the body will lead to dynamic changes in its concentration and it is difficult to simulate the real situation with *in vitro* experiments, adding difficulties to the evaluation of human applicability and effectiveness of the conclusions. Accurate and reliable measurements of exposure to tobacco products are essential for identifying and confirming patterns of tobacco product use and for assessing their potential biological effects in both human populations and experimental systems. This is also a difficult research problem worldwide that needs further study.

## CONCLUSIONS

Periodontal disease is one of the major dental pathologies and it is also a multifactorial disease that involves microbial challenge and host responses. Cigarette smoking is a well-established risk factor for periodontal disease. Both active and passive smoking could aggravate the development of periodontal disease. Tobacco can directly damage periodontal ligament cells, accelerate the loss of alveolar bone and negatively affect the remodeling of alveolar bone during implantation and orthodontic treatment. Lower concentrations of CSE could promote the biofilm formation of the bacteria-related periodontal disease and have the potential to elicit a stress reaction, thus potentially working as an environmental modulating factor for bacterial metabolism and survival.

From the evidence available at present, tobacco significantly increases the prevalence of oral cancer and aggravates the damage from periodontal disease. But the evidence that periodontal disease may be related to the prevalence of oral cancer is limited. The *in vitro* experiments are not sufficient to link the effects of tobacco to periodontal disease given the complexity of host response. The mechanisms of tobacco in the exacerbation and progression of periodontal disease and the specific molecular mechanisms linking periodontal disease and oral cancer need to be further explored.

## CONFLICTS OF INTEREST

Authors have completed and submitted the ICMJE Form for Disclosure of Potential Conflicts of Interest and none was reported.
